# Risk of complications with prolonged operative time in morbidly obese patients undergoing elective total knee arthroplasty

**DOI:** 10.1186/s42836-022-00162-3

**Published:** 2023-02-02

**Authors:** Raman Mundi, Nicholas Nucci, Jesse Wolfstadt, Daniel Pincus, Harman Chaudhry

**Affiliations:** 1 Sunnybrook Holland Orthopaedic and Arthritic Centre, Division of Orthopaedic Surgery, University of Toronto, Toronto, ON M4Y 1H1 Canada; 2 Division of Orthopaedic Surgery, University of Ottawa, Ottawa, ON K1H 8L6 Canada; 3 Mount Sinai Hospital, Division of Orthopaedic Surgery, University of Toronto, Toronto, ON M5G 1X5 Canada

**Keywords:** Obesity, Total knee arthroplasty (TKA), Complications, Operative time

## Abstract

**Background:**

Patients with a high body-mass index (BMI) are at increased risk for significant complications after total knee arthroplasty (TKA). We explored whether operative time is a modifiable risk factor for infectious and thromboembolic complications.

**Methods:**

A retrospective observational cohort study of the ACS-NSQIP registry, including all patients who underwent primary TKA (2015–2018), and were morbidly obese (BMI 40 kg/m^2^ or greater) was performed. We created four categories of operative time in minutes: less than 60, 60–90, 91–120, and greater than 120. The association of prolonged operative time with superficial/deep surgical site infection (SSI), DVT and PE within 30 days postoperatively was evaluated using multivariate logistic regression.

**Results:**

34,190 patients were included (median age 63 [IQR 57–68], mean BMI of 44.6 kg/m^2^ [SD 4.4]). The majority of patients had an operative time between 60–90 mins (*n* = 13,640, 39.9%) or 91–120 mins (*n* = 9908, 29.0%). There was no significant association between longer operative time and superficial/deep/organ-space SSI or PE. DVT risk was significantly increased. Patients with time exceeding 120 mins had nearly 2.5 greater odds of DVT compared to less than 60 minutes (OR 2.47, 95% CI: 1.39–4.39, *P* = 0.002). Odds of DVT were 1.73 times greater in those with time of 91–120 mins (OR 1.73, 95%CI: 0.98–3.05, *P* = 0.06).

**Conclusion:**

Early infection and thromboembolic complications with prolonged operative time in morbidly obese patients remain low. We did not identify a significant association with increased operative time and superficial/deep SSI, or PE. There was a significantly increased risk for deep vein thrombosis with prolonged operative time.

## Background

With approximately 800,000 procedures performed across North America per year, elective total knee arthroplasty (TKA) is a mainstay in the end-stage management of degenerative joint disease [1, 2]. It is anticipated that the annual number of knee replacements performed will exceed three million in the United States alone by 2030 [3].

Although TKA is generally a successful procedure in reducing pain and restoring patient function, certain patient populations are at heightened risk for significant medical and surgical complications [4–8]. In particular, it has been abundantly demonstrated that morbidly obese patients with a body mass index (BMI) of 40 kg/m^2^ or greater are at increased risk for several complications, including readmission, all-cause revision surgery, wound complications, prosthetic joint infections (PJI), and deep vein thrombosis (DVT) [9–12]. It has therefore been suggested that morbidly obese patients have their BMI optimized prior to elective joint surgery to reduce complication risk [10]. Unfortunately, substantial weight loss can be a difficult and unrealistic goal for many patients suffering from disabling knee arthritis. As an alternative, gastric bypass surgery prior to knee replacement has also been proposed and extensively explored. However, gastric bypass has inherent risks and the evidence evaluating the efficacy of bypass surgery for reducing complications after TKA remains questionable and conflicting [13–15]. There are also ethical factors to consider in denying such patients surgery, as total knee arthroplasty has been shown to confer similar functional benefits to obese patients as to non-obese patients [16].

A more feasible approach for risk mitigation in morbidly obese patients would be to identify relatively easy modifiable prognostic factors, such as operative time. Prolonged operative time during TKA has consistently been shown to increase the rate of complications, such as readmission, reoperation, wound complications, and infections [17–20]. A broad range of thresholds has been promoted as optimal time targets, ranging from 80 to 120 mins as a general rule for all arthroplasty patients [17, 20].

Unfortunately, higher BMI predisposes patients to prolonged operative time during TKA, given the technical challenges of operating on such patients [18, 21]. To our knowledge, however, no study has evaluated the risk of complications associated with prolonged operative time in morbidly obese patients (BMI ≥ 40 kg/m^2^). The risk of complications with increasing operative time may be disproportionately greater in this patient population as compared to the general arthroplasty cohort. As such, optimal operative time targets that reduce complications may be different.

To that end, we conducted a cohort study of high BMI patients (BMI ≥ 40 kg/m^2^) undergoing primary elective TKA, using the American College of Surgeons National Surgical Quality Improvement Program registry (ACS-NSQIP). Our objectives were two-fold: (1) To determine the overall incidence of major 30-day complications with prolonged operative time, including superficial surgical site infection (SSI), deep/organ-space SSI, deep vein thrombosis (DVT), and pulmonary embolism (PE); and (2) to determine if prolonged operative time was associated with an increased risk of these complications using multivariable regression analysis to control for confounding variables.

## Methods

### Study design

This study was a retrospective observational cohort study of the ACS-NSQIP registry, which is an international quality improvement program for surgical subspecialties. It is comprised of over 700 participating hospitals, largely from North America. Surgical patients were randomly sampled and outcomes at 30 days postoperatively were collected by trained reviewers.

### Inclusion criteria

We included all patients from the ACS-NSQIP registry between 2015 to 2018 who met the following inclusion criteria: (1) Underwent primary total knee arthroplasty as identified using the current procedural terminology code 27447; (2) Were morbidly obese as defined by a BMI of 40 kg/m^2^ or greater; and (3) Were documented as having an elective procedure. BMI was calculated for each patient based on their recorded weight and height.

### Data collection

We collected baseline demographic and comorbidity data including age, gender, American Society of Anesthesia (ASA) Score, smoking status, diabetic history, steroid use, and preoperative functional status. We also collected baseline treatment data including the year of surgery, discharge destination, and operative time. Operative time was converted into a categorical variable consisting of the following four categories: less than 60 mins, 60 to 90 mins, 91 to 120 mins, and greater than 120 mins. We selected these time categories based on the above-mentioned evidence, which has previously suggested optimal time targets for arthroplasty surgery ranging from 80 to 120 mins [17, 20].

Data were also collected on major 30-day complications including superficial SSI, deep/organ-space SSI, DVT, and PE. As per the NSQIP definitions, superficial SSI comprises any infection of the skin or subcutaneous tissue, whereas deep/organ-space SSI includes infections deep into the subcutaneous tissue. Deep venous thrombosis was defined as any thrombus warranting treatment and confirmed with imaging such as a duplex venogram or computed tomography (CT) scan. Pulmonary embolism was defined as a new clot in a pulmonary artery confirmed with a positive CT scan, transesophageal echocardiography, pulmonary arteriogram, CT angiogram, or high probability ventilation-perfusion scan.

### Data analysis

We present baseline and outcome data using descriptive statistics, including frequencies with associated percentages for categorical data and means or medians with appropriate measures of dispersion (standard deviation, interquartile range) for continuous variables.

The primary analysis was conducted using multivariable logistic regression models for each of the four major complications (dependent variables). We included several prognostic variables in the regression models to control for confounding, including age (< 59 *vs.* 60–69, 70–79, ≥ 80), gender (male *vs.* female), year of surgery (2015 *vs.* 2016, 2017, 2018), ASA score (1/2 *vs.* 3/4), smoking status (yes *vs.* no), diabetes status (yes *vs.* no), steroid use (yes *vs.* no), BMI (40–49 *vs.* 50–59, ≥ 60), discharge destination (home *vs.* other), functional status (independent *vs.* dependent/partially dependent), and operative time (< 60 mins *vs.* 60–90, 91–120, >120 mins). A preliminary assessment of outcome rates ensured each model would not be overfitted, with a minimum threshold of one confounder variable for every ten events.

All statistical analyses were done using IBM SPSS (Version 27). A *P* value of less than 0.05 was deemed as significant.

## Results

The final study included 34,190 patients that underwent primary elective TKA between 2015 and 2018. The cohort had a median age of 63 (IQR 57–68), 72% was female (*n* = 24,629), and had a mean BMI of 44.6 kg/m^2^ (SD 4.4). Baseline characteristics according to operative time are presented in Table 1.

**Table 1:** Baseline characteristics

Characteristic	Operative Time			
	< 60 mins (*n* = 4554)	60–90 mins (*n* = 13,640)	91–120 mins (*n* = 9908)	> 120 mins (*n* = 6088)
**Age**				
Median IQR	63 (57–69)	63 (57–69)	62 (56–68)	61 (55–67)
**Gender**				
Female (%)	3478 (76.4%)	10,145 (74.4%)	6936 (70.0%)	4066 (66.8%)
**BMI**				
Mean (SD)	44.4 (4.2)	44.5 (4.3)	44.7 (4.5)	44.9 (4.6)
**ASA Grade**				
3–4 (%)	3676 (80.7%)	10,700 (78.4%)	7822 (78.9%)	4766 (78.4%)
**Smoker **(%)	426 (9.4%)	1117 (8.2%)	860 (8.7%)	586 (9.6%)
**Diabetic **(%)	1362 (29.9%)	3838 (28.1%)	2793 (28.2%)	1646 (27.0%)
**Steroid Use **(%)	145 (3.2%)	448 (3.3%)	372 (3.8%)	207 (3.4%)
**Preoperative Functional Status**				
Independent (%)	4476 (98.3%)	13,393 (98.2%)	9748 (98.4%)	5930 (97.4%)
**Discharge Destination**				
Home (%)	3721 (81.7%)	11,055 (81.0%)	7567 (76.4%)	4171 (68.5%)

The majority of patients had an operative time between 60–90 mins (*n* =13,640, 39.9%) or 91–120 mins (*n* = 9908, 29.0%). Relatively fewer patients had a surgical time of less than 60 mins (*n* = 4554, 13.3%) or greater than 120 mins (*n* = 6088, 17.8%).

### Complications

The overall complication rate was low across all operative time cohorts (Table 2). With the exception of superficial SSI, all complications had a less than 1% incidence. The unadjusted relative risk of DVT was three times greater in the cohort with operative time exceeding 120 mins compared to those with operative time of less than 60 mins (Absolute Risks: 0.9% *vs.* 0.3%, respectively).

**Table 2:** Complication rates

Characteristics	Operative Time			
	< 60 mins (*n* = 4554)	60–90 mins (*n* = 13,640)	91–120 mins (*n* = 9908)	> 120 mins (*n* = 6088)
**Superficial SSI**	46 (1%)	128 (0.9%)	87 (0.9%)	67 (1.1%)
**Deep/Organ SSI**	25 (0.5%)	60 (0.4%)	70 (0.7%)	44 (0.7%)
**DVT**	15 (0.3%)	67 (0.5%)	59 (0.6%)	56 (0.9%)
**Pulmonary Embolism**	21 (0.5%)	83 (0.6%)	44 (0.4%)	43 (0.7%)

In the regression analyses, there was no statistically significant association between longer operative time and superficial SSI, deep/organ-space SSI, or pulmonary embolism (Table 3). Older age, steroid use, smoking and higher BMI were associated with increased odds of superficial SSI. Male gender, non-independent functional status, and higher BMI were associated with increased risk of deep/organ-space SSI. Functional status and a discharge destination other than home were associated with increased odds of pulmonary embolism.

**Table 3:** Multivariable logistic regression analyses of complications risk

**Risk Factor**	**Superficial SSI**	**Deep/Organ-Space SSI**	**Pulmonary Embolism**	**Deep Venous Thrombosis**
	**OR (95% CI)**	**OR (95% CI)**	**OR (95% CI)**	**OR (95% CI)**
**Operative Time**				
< 60 mins (ref)	1	1	1	1
60–90 mins	0.93 (0.67–1.31)	0.78 (0.49–1.24)	1.30 (0.80–2.10)	1.49 (0.85–2.61)
91–120 mins	0.85 (0.59–1.22)	1.23 (0.78–1.95)	0.89 (0.53–1.51)	1.73 (0.98–3.05)**
>120 mins	1.08 (0.74–1.58)	1.21 (0.74–2.00)	1.30 (0.77–2.21)	2.47 (1.39–4.39)*
**Age**				
< 59 (ref)	1	1	1	1
60–69	1.20 (0.93–1.55)	1.05 (0.77–1.45)	0.97 (0.69–1.36)	0.94 (0.68–1.30)
70–79	1.37 (0.99–1.90)	1.12 (0.73–1.71)	1.23 (0.82–1.85)	0.80 (0.52–1.23)
80+	2.37 (1.25–4.51)*	1.96 (0.83–4.63)	1.20 (0.47–3.05)	1.45 (0.65–3.22)
**Female**	1.01 (0.79–1.30)	0.63 (0.47–0.85)	0.88 (0.64–1.21)	0.91 (0.67–1.25)
**BMI**				
40–49 (ref)	1	1	1	1
50–59	2.07 (1.54–2.77)*	2.54 (1.79–3.61)*	1.18 (0.76–1.85)	0.63 (0.37–1.10)
≥ 60	2.28 (1.06–4.87)*	1.59 (0.50–5.02)	0.47 (0.07–3.36)	0.83 (0.21–3.38)
**ASA**				
1/2 (ref)	1	1	1	1
3/4	1.12 (0.83–1.50)	1.18 (0.81–1.73)	1.08 (0.74–1.56)	1.20 (0.82–1.75)
**Smoker**	1.52 (1.08–2.15)*	0.91 (0.54–1.52)	0.91 (0.53–1.56)	0.65 (0.36–1.18)
**Non-smoker (ref)**	1	1	1	1
**Diabetes**	1.06 (0.83–1.35)	1.03 (0.75–1.40)	0.75 (0.53–1.05)	1.03 (0.75–1.41)
**Non-diabetic (ref)**	1	1	1	1
**Steroid**	1.87 (1.18–2.96)*	0.91 (0.40–2.05)	1.20 (0.59–2.44)	1.61 (0.87–2.97)
**No Steroid (ref)**	1	1	1	1
**Functionally Non-independent**	1.28 (0.65–2.51)	2.37 (1.20–4.69)*	2.03 (1.03–4.01)*	1.49 (0.69–3.19)
**Independent (ref)**	1	1	1	1
**Not Discharged to Home**	1.13 (0.87–1.47)	1.00 (0.71–1.41)	2.24 (1.65–3.05)*	2.28 (1.68–3.09)*
**Discharged Home (ref)**	1	1	1	1

* *P* < 0.05, ** *P* = 0.06

The risk of DVT was significantly associated with prolonged operative time. Patients with operative time exceeding 120 mins had nearly 2.5 greater odds of DVT compared to those with operative time of less than 60 mins (OR 2.47, 95% CI: 1.39–4.39, *P* = 0.002). The odds of DVT were 1.73 times greater in those with operative time of 90–120 mins. However, the lower limit of the 95% confidence interval marginally crossed the line of non-significance (OR 1.73, 95% CI: 0.98–3.05, *P* = 0.06).

## Discussion

In this observational cohort study of 34,190 patients with high BMI, it was demonstrated that the overall rate of infectious and thromboembolic complications at 30-days was low. Prolonged operative time did not increase the risk of superficial and deep wound infections, or pulmonary embolism. However, there was an increased risk of deep vein thrombosis in patients with longer operative times.

Several ACS-NSQIP registry studies evaluating complications in total joint replacement patients have been previously published. Duchman and colleagues evaluated the effect of operative time on complications in a general cohort of 99,444 joint replacement patients (THA and TKA). In their multivariate regression analyses, they identified prolonged operative time to be significantly associated with the composite outcomes of "overall complications" (OR 1.15, 95% CI: 1.05–1.26) and "wound complications" (OR 1.44, 95% CI: 1.21–1.71). They did conduct a univariate analysis for individual complications, in which they identified that prolonged operative time increased the risk of both superficial and deep wound infection, but not DVT or PE [17].

In a similar study evaluating a more remote cohort of 165,474 joint replacement patients (THA and TKA) by Bohl *et al*., it was demonstrated that every 15-min increase in operative time increases surgical site infection risk by 9% (RR 1.09, 95% CI: 1.07–1.12). They did not, however, find an increased risk of DVT (RR 0.99, 95% CI: 0.97–1.02) or PE (RR 1.01, 95% CI: 0.98–1.05). In a subgroup analysis of only TKA patients, the statistical significance of these findings did not change [22].

The findings of the above studies appear to conflict with the findings of our current study, in which we identified no significant increase in the risk of 30-day SSI (superficial or deep), but a significantly increased risk of DVT. Although there were subtle differences in analytic approaches (composite *vs.* individual outcomes, time cut-offs, regression model variables, *etc.*), we speculate that the primary contributor to the discrepancy in our study conclusions is due to the difference in our study populations. Our study included only patients undergoing TKA, whereas the primary analyses in the prior studies combined TKA and THA patients. Furthermore, our study exclusively evaluated patients that were morbidly obese, with a mean BMI of 44.6 kg/m^2^. The mean BMI was 31.7 kg/m^2^ in the study by Duchman and colleagues, and only 55% of patients had a BMI ≥ 40 kg/m^2^ in the study by Bohl *et al*. [17, 22]. Patients with high BMI may have an inherently increased risk of infection at baseline, and prolonged operative time have less effect on infection risk. For instance, the absolute risk of infection at baseline (operative time < 60 mins) was substantially higher in our study population compared to the study by Bohl *et al*. (Superficial SSI: 1% *vs.* 0.5%; Deep/Organ-Space SSI: 0.5% *vs.* 0.2%). Finally, we found that increased operative time was associated with DVT risk in obese patients undergoing TKA, whereas the above-mentioned studies did not identify an association. A theoretical explanation for this DVT risk may be that high BMI patients have excess soft tissue in the posterior calf and thigh, which leads to increased compression and venous outflow obstruction during knee flexion. Under these circumstances, prolonged operative time may lead to increased DVT risk. Interestingly, however, increased BMI itself has not always been shown to increase the risk of DVT after primary TKA [23].

Importantly, the ACS-NSQIP studies only evaluated outcomes at 30-day and this limited follow-up likely underestimated the true incidence of complications. In a single-centre retrospective cohort study of 17,342 joint replacement patients, Wang *et al*. identified an overall 90-day surgical site infection risk of 2.1% and a 1-year PJI risk of 1.4%, in patients with operative time exceeding 90 min. Every 20-min increase in operative time resulted in a 35% increased risk of SSI and a 25% increased risk of PJI. However, the mean BMI in this cohort was approximately 30 kg/m^2^ [24]. We are unaware of any studies evaluating exclusively high BMI patients with such longer-term follow-up.

The current study has several strengths. To our knowledge, it's the first large database study to evaluate the effect of operative time on infectious and thromboembolic complications in high BMI patients undergoing primary TKA. Secondly, we used a recent cohort of patients from 2015 to 2018, in contrast to other recent studies that have assessed more remote cohorts. Finally, by exclusively evaluating high BMI patients, we may have controlled for a confounding variable spuriously correlating operative time to acute infection risk. Rather than prolonged operative time resulting in higher infection risk, perhaps increased case complexity (such as high BMI) results in both longer operative time and infection risk.

Our study also has some limitations. It is retrospective in nature and only includes outcomes up to 30-days postoperatively. Furthermore, given that NSQIP is a large, multi-national database with limited data collection, several important prognostic factors that could influence the outcomes of interest were not collected, such as antibiotic and anticoagulation protocols. It must also be recognized with any large dataset analysis, that statistical significance does not always correlate with clinical significance. As such, whether the increased risk of DVT with prolonged operative time is of clinical importance, is subject to debate.

## Conclusion

The overall infection and thromboembolic complication rates with prolonged operative time in morbidly obese patients remain low at 30 days postoperatively. There appears to be a significantly increased risk for deep vein thrombosis with prolonged operative time, but whether this is clinically significant remains debatable. Future studies are warranted to corroborate these findings in high BMI patients. Fortunately, despite the technical demands of performing a TKA in obese patients, a 90-min operative time target is one that is feasible for most surgeons and for the majority of cases.

## Data Availability

The dataset supporting the conclusions of this article is available in the ACS-NSQIP repository, https://www.acsdataplatform.com/login.

## Abbreviations

BMI: Body-mass index
TKA: Total knee arthroplasty
SSI: Surgical site infection
THA: Total hip arthroplasty
PJI: Prosthetic joint infection
DVT: Deep vein thrombosis
PE: Pulmonary embolism
ASA: American Society of Anesthesia
CT: Computed tomography
